# Novel Cholesterol-Based Cationic Lipids as Transfecting Agents of DNA for Efficient Gene Delivery

**DOI:** 10.3390/ijms16035666

**Published:** 2015-03-11

**Authors:** Jia Ju, Meng-Lei Huan, Ning Wan, Hai Qiu, Si-Yuan Zhou, Bang-Le Zhang

**Affiliations:** Department of Pharmaceutics, School of Pharmacy, Fourth Military Medical University, Xi’an 710032, China; E-Mails: yqyhjj@fmmu.edu.cn (J.J.); hmlkuohu@fmmu.edu.cn (M.-L.H.); yaoji1316@fmmu.edu.cn (N.W.); yaojishi@fmmu.edu.cn (H.Q.); zhousy@fmmu.edu.cn (S.-Y.Z.)

**Keywords:** cholesterol, cationic liposome, lysine, histidine, synthesis

## Abstract

The design, synthesis and biological evaluation of the cationic lipid gene delivery vectors based on cholesterol and natural amino acids lysine or histidine are described. Cationic liposomes composed of the newly synthesized cationic lipids **1a** or **1b** and neutral lipid DOPE (1,2-dioleoyl-l-α-glycero-3-phosphatidyl-ethanolamine) exhibited good transfection efficiency. pEGFP-N_1_ plasmid DNA was transferred into 293T cells by cationic liposomes formed from cationic lipids **1a** and **1b**, and the transfection activity of the cationic lipids was superior (**1a**) or parallel (**1b**) to that of the commercially available 3β-[*N*-(*N*',*N*'-dimethylaminoethyl)-carbamoyl] cholesterol (DC-Chol) derived from the same cholesterol backbone with different head groups. Combined with the results of agarose gel electrophoresis, transfection experiments with various molar ratios of the cationic lipids and DOPE and N/P (+/−) molar charge ratios, a more effective formulation was formed, which could lead to relatively high transfection efficiency. Cationic lipid **1a** represents a potential agent for the liposome used in gene delivery due to low cytotoxicity and impressive gene transfection activity.

## 1. Introduction

Gene therapy plays an important role in the treatment of inherited and acquired diseases, such as cancer, cardiovascular diseases, AIDS and autoimmune disorders. The success of gene therapy relies on finding efficient and safe vectors for gene delivery [1,2,3,4]. Gene delivery vectors are classified into viral and non-viral ones. Among them, viral vectors are an efficient means for gene delivery, but some side effects, including immunogenicity and biological safety, limit application in clinical trials [5,6]. For these reasons, non-viral vectors, such as cationic lipids, cationic polymers and peptides, represent an attractive, alternative approach to gene delivery [7,8,9]. Liposomes based on cationic lipids have been favored for many potential advantages compared with other non-viral vectors, which, in general, exhibit excellent biocompatibility, low immunogenicity, low toxicity and large nucleic acid carrying capacity.

**Figure 1:**
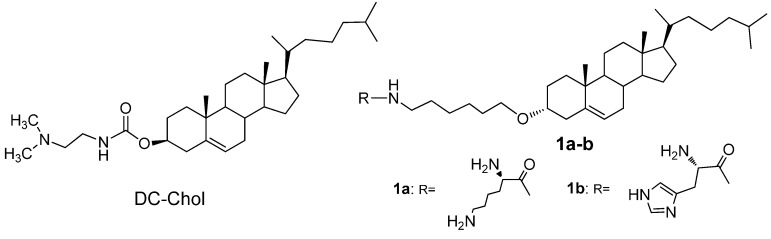
Molecular structures of cholesterol-derived lipid 3β-[*N*-(*N*',*N*'-dimethylaminoethyl)-carbamoyl] cholesterol (DC-Chol) and the designed lipids.

Cationic lipids are commonly composed of three parts, including positive-charged polar head group, a linker and a hydrophobic tail [10]. The head groups consist of amines or those of an extended formation, such as methylamine, ethylamine, propylamine and polyamine [11,12,13], and the hydrophobic tails often contain saturated or mono-unsaturated fatty acid chains containing typically 14 to 18 carbon atoms or a rigid cholesteryl moiety [14,15]. The cholesterol-based cationic lipid has been used as the major hydrophobic domain of liposomes for gene delivery due to being less toxic than other cationic lipids [16]. Many cholesterol-based cationic lipids have been synthesized, among which 3β-[*N*-(*N*',*N*'-dimethylaminoethyl)-carbamoyl] cholesterol (DC-Chol, Figure 1) showed efficient transfection in gene delivery and was used as the commercially available liposome reagent [17,18,19,20].

The positive-charged head group and linker are also important factors related to successful gene delivery. A common empirical approach to improve transfection efficiency and to reduce cellular toxicity focusses on structural modifications of the head group functionality, in which the cholesterol backbone is linked to various polyamine cationic head groups [11,12,13,21,22]. A basic amino acid is a good choice as head groups in cationic lipids for its positive-charged profile. Many basic amino acid derivatives have been introduced into gene delivery carriers and exhibited good gene transfection properties [23,24,25,26,27]. The hydrophobic domain and positive-charged head group of cationic lipids can be linked via carbamate, amide, ester or ether bonds. Li *et al*. [12] have reported new cationic lipids by incorporating amino acids directly via ester bonds. Meanwhile, Sheng *et al*. [28] synthesized and characterized a series of cholesterol and amino acid-based cationic lipids through ester and ether linkages. It has been noticed that both ether- and amide-linked lipids are more stable than the carbamate- or ester-linked lipids and provided an improved gene transfer efficiency [29,30,31,32,33]. The linker of amide and ether bonds to cationic lipids might be attributed to a stable and highly efficient gene transfer system.

For safe and efficient gene delivery, developing new gene vectors with low cytotoxicity and high gene transfection performance is a major task. The cytotoxicity and gene transfection efficiency strongly depend on the molecular backbone of the vectors. Thus, selecting appropriate molecular building blocks and linkers to construct low toxic and highly efficient gene vectors is important in gene therapy. In this paper, we designed and synthesized two novel cationic lipids based on a cholesterol backbone (Figure 1), where the cholesterol was linked to positive-charged basic amino acid residues (lysine and histidine) via a stable ether linked to the cholesterol backbone and an amide linked to the amino acid. The biological behaviors of both lysine- and histidine-modified cholesterol cationic lipids were evaluated and exhibited high delivery efficiency and low toxicity, indicating them as potential gene delivery vectors.

## 2. Results and Discussion

### 2.1. Chemistry

**Figure 2:**
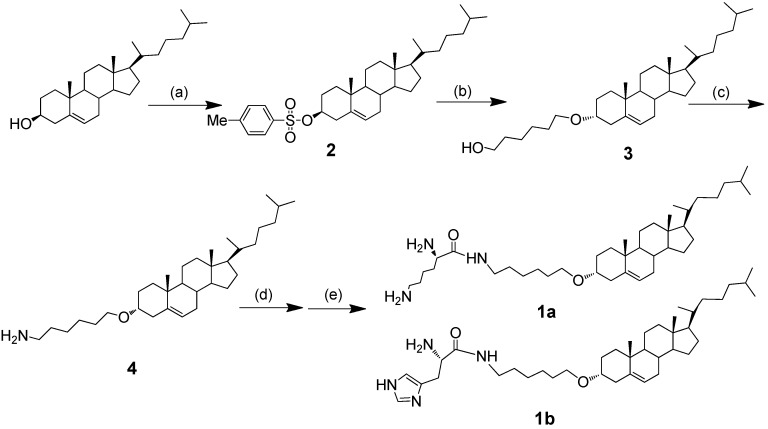
Synthetic route of the novel cationic lipids derived from cholesterol. Reagents and conditions: (**a**) *p*-tosyl chloride (*p*-TsCl)/pyridine/CHCl_3_, 0 °C, 1 h, rt, 23 h; (**b**) 1,6-hexanediol, anhydrous dioxane, reflux, 7 h; (**c**) (i) hydrazoic acid (HN_3_), triphenylphosphine (PPh_3_), diisopropyl azodicarboxylate (DIAD), 0 °C, overnight, 50 °C, 12 h; (ii) PPh_3_, 50 °C, 12 h; (iii) H_2_O, rt, overnight; (**d**) Boc-Lys(Boc)-OH or Boc-His(Boc)-OH, *N*,*N*'-dicyclohexylcarbodiimide (DCC)/*N*-hydroxysuccinimide (NHS), THF, 0 °C, 7 h; (**e**) TFA, CH_2_Cl_2_, 0 °C, 2 h.

Cationic lipids are based on a cholesterol backbone, where the cholesterol is linked to positive-charged basic amino acid residues via ether linked to the cholesterol backbone and amide linked to the amino acid (Figure 2). The cationic lipids **1a** and **1b** have been synthesized by reacting the precursor **4** with corresponding amino-protected lysine and histidine. Compound **4** was synthesized from natural cholesterol through three steps. Compound **2** was synthesized in an 86.6% yield after recrystallization by modifying the reported method [14]. Then, cholesterol tosylate **2** and 1,6-hexanediol (7 eq) in anhydrous dioxane were refluxed for 7 h to afford Compound **3** in a 78.2% yield. Then, Compound **3** reacted with hydrazoic acid (HN_3_) (1.3 eq, 1 M in THF), diisopropyl azodicarboxylate (DIAD) (1.25 eq) and triphenylphosphine (PPh_3_) (1.25 eq) in THF, affording **4** in a 65.4% yield. To introduce the polyamine cationic head group of lysine and histine residues, **4** was first condensated by reacting with Boc-Lys (Boc)-OH (1.2 eq) or Boc-His(Boc)-OH (1.2 eq), *N*,*N*'-dicyclohexylcarbodiimide (DCC) (3 eq) and *N*-hydroxysuccinimide (NHS) (1.2 eq) in THF, then deprotection with TFA at 0 °C for 2 h, followed by purification with column chromatography to give **1a** and **1b** in yields of 15.0% and 39.9% respectively. The synthetic route is shown in Figure 2. The chemical structure of the synthesized lipids was characterized by the ^1^H NMR and ESI-MS spectrum. The two cationic lipids showed the typical proton signal at chemical shifts of 2.51–0.67 ppm, resulting from the cholesterol lipid skeleton, and at 5.35 ppm (cholesterol double bond). The lipids bearing the lysine head group showed obvious peaks at 3.52–3.43 ppm, which were identified as typical proton signals of the –CH_2_– attached to the proton at the primary amino group of lysine. Meanwhile, **1b** lipids also showed their typical imidazole proton peaks at 7.55 and 6.84 ppm. The spectrum identified the synthesized cholesterol cationic lipids, consistent with the targeted compounds.

### 2.2. Characterization of Neat Cationic Liposomes

**Figure 3:**
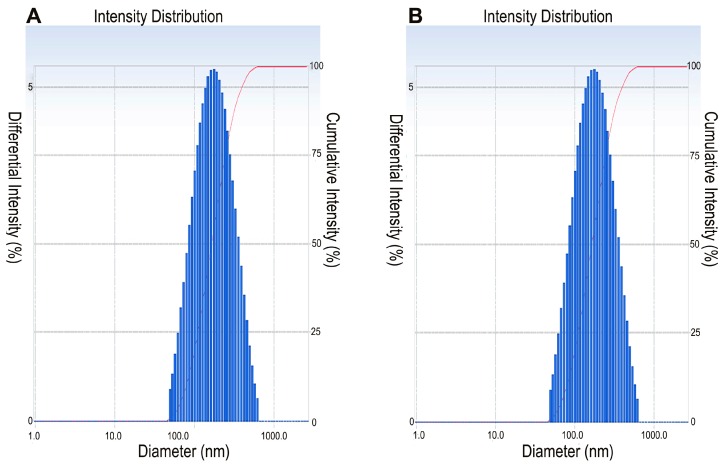
Particle size distribution of liposomes prepared from cationic lipid **1a** (**A**) and **1b** (**B**).

To form the cationic liposome formulation, neutral lipid DOPE (1,2-dioleoyl-L-α-glycero-3-phosphatidyl-ethanolamine) was used as the co-lipid. Liposomes could be conveniently prepared from cationic lipids **1a** or **1b** with DOPE at a molar ratio of 1:1 by first subjecting the films to the lipid mixtures. All lipids dispersed in deionized water easily and formed stable and clear liposomes. No precipitation or noticeable increase in turbidity was observed, even when stored at 4 °C under sterile conditions one month later. The particle size distribution and zeta potential of the liposome’s dispersion were determined at 25 °C by the dynamic light scanning method with the Delsa™ Nano C Particle Analyzer (Beckman Coulter, Brea, CA, USA) (Table 1, Figure 3). The results obtained from dynamic light scattering experiments are shown in Table 1. The particle size and zeta potential of the prepared cationic liposomes were 143.0 ± 1.7 nm and 23.7 ± 3.9 mV for **1a** and 144.5 ± 2.0 nm and 47.7 ± 4.1 mV for **1b**, respectively, indicating that the amino acid residues of cationic lipids showed little effect on the physical properties of liposomes.

**Table 1:** Particle size and zeta potential of cationic liposomes obtained from DLS measurements.

Cationic Lipid	Size	Polydispersity Index (PDI)	Zeta Potential
**1a**	143.0 ± 1.7 nm	0.260 ± 0.020	23.7 ± 3.9 mV
**1b**	144.5 ± 2.0 nm	0.289 ± 0.008	47.7 ± 4.1 mV

### 2.3. Gel Electrophoresis

**Figure 4:**
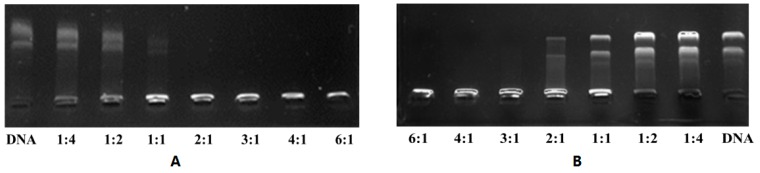
Electrophoretic gel patterns for lipoplex-associated pDNA in gel retardation using cationic lipids **1a** (**A**) or **1b** (**B**) at various N/P ratios. The N/P (+/−) molar charge ratio is indicated at the bottom of each lane.

Once negative DNA encountered the liposome containing cationic lipid, it might produce a complex due to attraction. To characterize the electrostatic binding interactions between the plasmid DNA and the mixed cationic liposomes (lipid:DOPE = 1:1) as a function of different N/P (+/−) molar charge ratios, we performed conventional electrophoretic gel retardation assays. pEGFP-N1 was used as the pDNA to test the binding ability. The results of the gel retardation assay indicated that the DNA binding ability of cationic liposomes increased with an increase in the N/P ratio. As shown in Figure 4, cationic lipids **1a** and **1b** were able to be retard the plasmid DNA at a N/P ratio of 2:1 (**1a**:DNA molar ratio of 1:1), and 3:1 (**1b**:DNA molar ratio of 1.5:1), respectively. Between the two cationic liposome formulations, more than 90% of the plasmid DNA got retarded at the N/P ratio of 1.0 with cationic lipid **1a**, giving a high performance for DNA binding. However, cationic lipid **1b** might achieve a N/P ratio of 2.0 to show efficient binding capacity, revealing that the structure of head groups is related to their DNA binding abilities.

### 2.4. Transfection Biology

#### 2.4.1. Optimization of the Ratio of Lipid and DOPE

**Figure 5:**
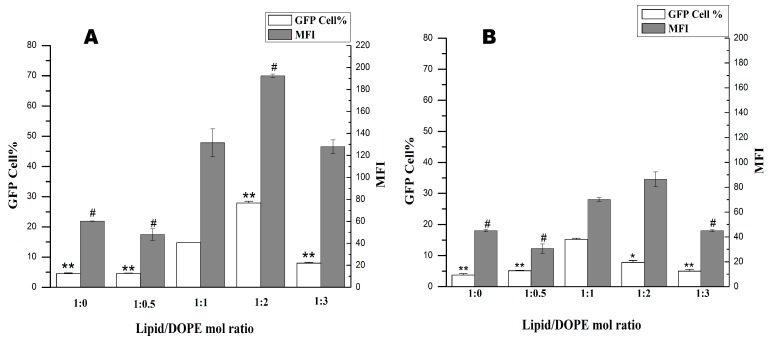
Transfection efficiencies of cationic lipids **1a** (**A**) and **1b** (**B**) with various compositions of DOPE (1,2-dioleoyl-l-α-glycero-3-phosphatidyl-ethanolamine) in 293T cells. Each bar value represents the mean ± SD of triplicate experiments. Data are expressed as the number of transfected cells and mean fluorescence intensity (MFI) as obtained from flow cytometry analysis. * *p* < 0.05, ** *p* < 0.01, compared with the number of transfected cells at a lipid:DOPE ratio of 1:1. ^#^ *p* < 0.05, compared with the mean fluorescence intensity at a lipid:DOPE ratio of 1:1.

**Figure 6:**
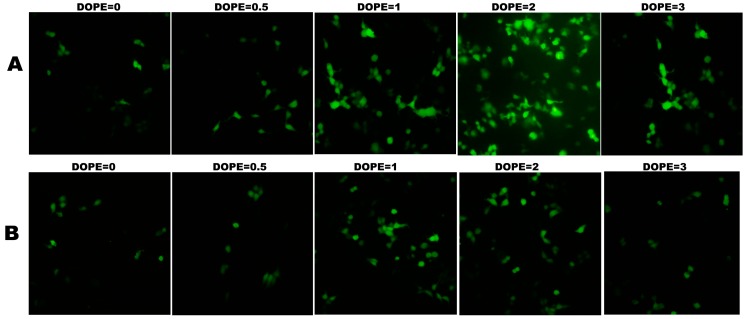
Expression of GFP in 293T cells under a fluorescence microscope using **1a** (**A**) and **1b** (**B**) as cationic lipids at the different molar ratios of DOPE.

Neutral lipids also played an important role when forming high activity cationic liposome [16,34,35]. Therefore, the ratios of cationic lipids **1a** or **1b** and DOPE in liposome formulations were optimized with N/P ratios. Both the amount of positive transfected cells and the mean fluorescence intensity (MFI) have been recorded for transfection efficiency evaluation. Gene transfections with various mol ratios of the cationic lipids (**1a** and **1b**) and neutral lipid DOPE were performed. As shown in Figure 5, when the DOPE molar ratio increased from one to two, significantly enhanced gene transfection activities were observed for lipid **1a**, whereas decreased transfection activities for **1b**. However, the transfection efficiency decreased with the further increase of the DOPE molar ratio to three, both for **1a** and **1b**. In contrast, when no co-lipid DOPE or less DOPE (molar ratio of 0.5) was added, drastically decreased transfection efficiency was found both for **1a** and **1b**, less effective both in terms of the number of transfected cells and MFI. The results indicated that the optimized lipid/DOPE ratios of **1a** and **1b** were 1:2 and 1:1, respectively. When compared with the transfection efficiencies at their optimal lipid/DOPE ratio, lipid **1a** was found to be a superior transfecting agents, which has been confirmed by fluorescence microscopy (Figure 6).

#### 2.4.2. Optimization of N/P Charge Ratio

**Figure 7:**
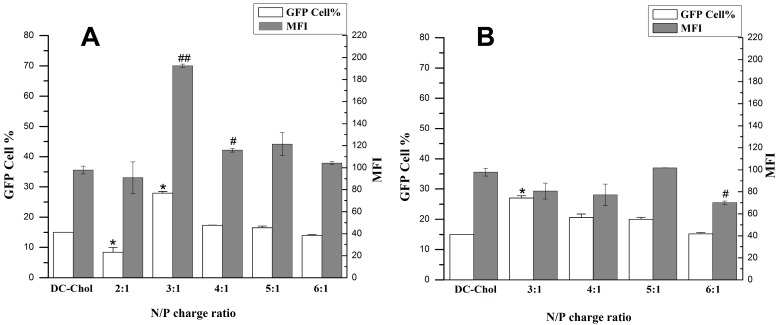
Transfection efficiencies of cationic lipids **1a** (**A**) and **1b** (**B**) with the optimum compositions of DOPE at the various N/P ratios in 293T cells. Each bar value represents the mean ± SD of triplicate experiments performed on the same day. Data are expressed as the number of transfected cells and MFI as obtained from flow cytometry analysis. * *p* < 0.05, compared with the number of transfected cells of DC-Chol. ^#^ *p* < 0.05, ^##^ *p* < 0.01, compared with the mean fluorescence intensities of DC-Chol.

**Figure 8:**
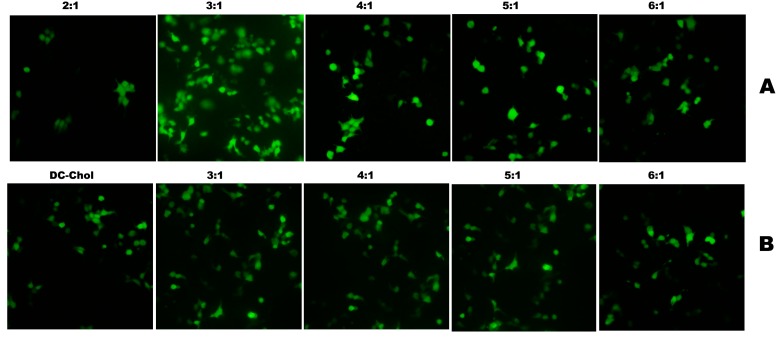
Expression of GFP in 293T cells under a fluorescence microscope using cationic lipids **1a** (**A**) and **1b** (**B**) with the optimum compositions of DOPE at the various N/P ratios in 293T cells.

After an optimized lipid/DOPE ratio was determined, we further measured the transfection efficiency at various N/P (+/−) molar charge ratios to seek the optimum transfection condition by using flow cytometry analysis and fluorescence microscope. The results indicated that the newly synthesized cationic lipid **1a** exhibited higher transfection efficiency than DC-Chol in optimum transfection conditions determined by flow cytometry analysis. The N/P ratio of three (**1a**:DNA molar ratio of 1.5:1) showed higher transfection efficiency, both in the number of transfected cells and the mean fluorescence intensity (MFI), than DC-Chol. Cationic lipid **1a** was able to transfect 30% of cells with an MFI of 190 at a N/P ratio of 3:1. While the transfection efficiencies of **1b** were similar or slightly superior to DC-Chol, both the number of transfected cells and the mean fluorescence intensity in a N/P ratio from 3:1 to 6:1. Cationic lipid **1b** could transfect approximately 25% of the cells with an MFI of nearly 80 at the same N/P ratio of 3:1 (Figure 7). All results could also be confirmed by GFP expression observed under a fluorescence microscope (Figure 8).

### 2.5. Cytotoxicity Assay

**Figure 9:**
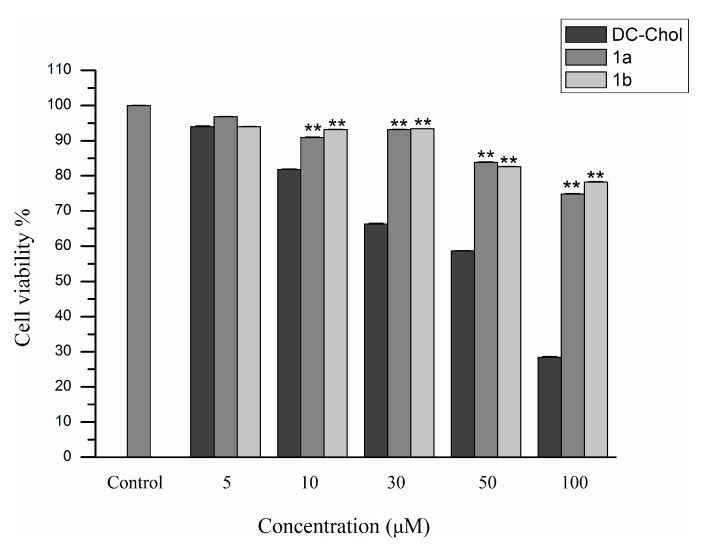
MTT assay-based cellular cytotoxicity of blank cationic liposome generated from **1a** and **1b** against 293T cells. Each bar value represents the mean ± SD (*n* = 6). ** *p* < 0.01, compared with the cell viability of DC-Chol.

Since favorable gene carriers should have low toxicity, the toxicity of cationic lipids **1a** and **1b** towards 293T cells was determined using the 3-(4,5-dimethylthiazole-2-yl)-2,5-diphenyltetrazolium bromide reduction assay following a literature procedure. The results were expressed as a percentage of cell viability with respect to a control corresponding to untreated cells. As shown in Figure 9, cationic lipids **1a** and **1b** showed much higher cell viabilities than that of the DC-Chol group against 293T cells. When the concentration of DC-Chol reached 100 μM, only 28% of cells were still alive. In contrast, both cationic lipids **1a** and **1b** were found to have low cytotoxicity, even up to 100 μM, much higher than the actual amount used for gene transfection, which indicated that these two cationic lipids were both safe and highly efficient.

## 3. Experimental Section

### 3.1. Materials

DC-Chol was purchased from Sigma (St. Louis, MO, USA). DOPE was purchased from Fluka (Buchs, Switzerland). Cell culture media, Opti-MEM and fetal bovine serum (FBS) were purchased from Gibco (Carlsbad, CA, USA). 293T (human embryonic kidney cells) cell lines were procured from the Culture Collection of the Chinese Academy of Science (Shanghai, China). Cells were grown at 37 °C in Dulbecco’s Modified Eagle’s Medium (DMEM) with 10% FBS in a humidified atmosphere containing 5% CO_2_. pEGFP-N1, which encodes the enhanced green fluorescence protein (GFP) under a CMV promoter, was bought from Shanghai GenePharma Co., Ltd. (Shanghai, China). The progress of the reactions was monitored by thin-layer chromatography on silica gel plates from Kehao (Xi’an, China). Column chromatography was performed with silica gel (100–200 mesh, Kehao). Cholesterol was purchased from Kehao and used without further purification.

### 3.2. Synthesis of Cationic Lipids

#### 3.2.1. Cholest-5-en-3β-tosylate (**2**)

Cholest-5-en-3β-tosylate (**2**) was prepared as previously described with a minor modification [14]. In brief, to an ice-cooled solution of cholesterol (10.0 g, 25.86 mmol) in dry pyridine (20 mL) and dry chloroform (20 mL), *p*-tosyl chloride (6.78 g, 38.78 mmol) and a catalytic amount of 4-dimethylaminopyridine (DMAP) were added. The reaction mixture was allowed to stir at 0 °C for 1 h and further stirred 23 h at room temperature. The solvent was then evaporated. Chloroform (120 mL) was added to the reaction mixture and washed with 1 N HCl (3 × 40 mL) and water (50 mL). The organic layer was separated and dried over anhydrous Na_2_SO_4_. The solvent was evaporated, and the residue was recrystallized using acetone to afford cholest-5-en-3β-tosylate (**2**), 12.12 g. White solid; yield: 86.6%; mp: 133–134 °C; IR (KBr): 2948, 1356, 1190, 861, 814 cm^−1^; MS [*m*/*z*, ESI^+^] = 542 [M + H]^+^.

#### 3.2.2. Cholest-5-en-3β-hexane (**3**)

Cholest-5-en-3β-hexane (**3**) was synthesized according to a literature procedure with a minor modification [28]. Cholest-5-en-3β-tosylate (**2**) (5 g, 9.24 mmol) and 1,6-hexanediol (7.65 g, 64.71 mmol) were added to anhydrous dioxane (35 mL), and the mixture was refluxed for 7 h. The solution was cooled, and the solvent was removed under vacuum. The residue was dissolved in chloroform (100 mL) and washed with NaHCO_3_ (3 × 70 mL) and water (70 mL). The organic layer was separated and dried over anhydrous Na_2_SO_4_. Finally, the solvent was removed in a vacuum, and the residue was purified by column chromatography over silica with a mixture of petroleum ether and ethyl acetate (*v*/*v*, 3:1). White solid; yield: 78.2%; mp: 102–103 °C; MS [*m*/*z*, ESI^−^] = 485 [M − H]^−^.

#### 3.2.3. Cholest-5-en-3β-yl 6-Aminohexyl Ether (**4**)

A well-stirred mixture of cholest-5-en-3β-hexane (**3**) (2 g, 4.12 mmol) and triphenylphosphine (1.34 g, 5.12 mmol) in absolute THF (20 mL) was cooled to 0 °C, to which hydrazoic acid-chloroform (1 mmol/L, 5.36 mL, 5.36 mmol) was added. After stirring for 30 min, DIAD (diisopropyl azodicarboxylate, 1.02 mL, 5.12 mmol) was added dropwise. The mixture was stirred in an ice bath overnight and further for 12 h at 50 °C. Then, triphenylphosphine (1.34 g, 5.12 mmol) was added, and the solution was stirred at 50 °C for another 12 h. Then, water (8 mL) was added, and the solution was stirred overnight. Solvents were removed in a vacuum, and the residue was dissolved in CH_2_Cl_2_ and poured into 2 M hydrochloric acid (*v*/*v*, 1:1, 40 mL). The aqueous phase was washed with CH_2_Cl_2_ (3 × 50 mL). Then, 2 M NaOH was added until pH 11. The mixture was extracted with CH_2_Cl_2_ (3 × 60 mL), and the combined organic phase was washed with saturated NaHCO_3_ aqueous solution (3 × 60 mL) and dried with Na_2_SO_4_. The solvent was removed, and the product, **4**, was obtained by purifying on silica gel (CH_2_Cl_2_:MeOH:Et_3_N, 40:1:1). Pale yellow solid; yield: 65.4%; mp: 73–74 °C; MS [*m*/*z*, ESI^+^] = 487 [M + H]^+^; IR(KBr): 3409, 3000, 1377, 1109, 750 cm^−1^; ^1^H NMR (400 MHz, CDCl_3_) δ (ppm): 0.67 (s, 3H), 0.85 (d, 6H, *J* = 8.0 Hz), 0.91 (d, 3H, *J* = 7.2 Hz), 0.99 (s, 3H), 1.01–2.38 (m, 38H), 2.70 (t, 2H, *J* = 7.2 Hz), 3.08–3.14 (m, 1H), 3.43 (t, 2H, *J* = 4.4 Hz), 5.34 (d, 1H, *J* = 3.2 Hz). The structure of the obtained compound was consistent with the literature [36].

#### 3.2.4. General Method for Synthesis of the Lipids **1a** and **1b**

DCC (733 mg, 3.55 mmol), NHS (163 mg, 1.2 mmol) and Boc-Lys(Boc)-OH (492 mg, 1.42 mmol) or Boc-His(1-Boc)-OH (506 mg, 1.42 mmol) were dissolved in THF (25 mL) and allowed to stir at 0 °C for 30 min and further stirred 1 h at room temperature. Then, Compound **4** (575 mg, 1.18 mmol) was added and *N*,*N*’-dicyclohexylurea (DCU) removed by filtration after stirring 6 h at room temperature. The filtrate was evaporated and then dissolved in chloroform (30 mL). The chloroform solution was washed with distilled water (10 mL × 3) and dried with Na_2_SO_4_. After the chloroform solution was evaporated, the amino group-protected intermediates were obtained. The intermediates were deprotected with trifluoroacetic acid (12 mL) for 2 h at 0 °C, and lipids **1a** and **1b** were obtained with yields of 15.0%, and 39.9%, respectively. Cationic lipid **1a**: pale yellow solid; mp: 142–144 °C; IR (KBr): 3443, 3421, 3386, 1653, 1635, 1219, 771, 693, 556 cm^−1^; ^1^H NMR (CDCl_3_) δ (ppm) = 5.35 (t, *J* = 2.4 Hz, 1H), 4.30 (t, *J* = 6.4 Hz, 1H), 4.10–4.08 (m, 5H), 3.52–3.43 (m, 9H), 1.95–0.97 (m, 43H), 0.92–0.87 (m, 9H), 0.67 (s, 3H); MS [*m*/*z*, ESI^+^]: 615 [M + H]^+^. Cationic lipid **1b**: pale yellow solid, mp: 137–139 °C; IR(KBr): 3446, 3372, 1645, 1219, 1627, 1088, 771, 689, 556 cm^−1^; ^1^H NMR (CDCl_3_) δ = 7.55 (s, 1H), 6.84 (s, 1H), 5.34 (t, *J* = 2.4 Hz, 1H), 3.60–3.42 (m, 3H), 3.25–3.23 (m, 3H), 2.51–1.43 (m, 45H), 0.99–0.87 (m, 9H), 0.67 (s, 3H); MS [*m*/*z*, ESI^+^]: 624 [M + H]^+^.

### 3.3. Preparation of Liposomes

Individual lipid (15 μmol) and DOPE in desired molar ratios were dissolved in chloroform, and the thin films were made by evaporation of the organic solvent and kept under vacuum overnight to remove the residual solvent. Freshly deionized water (15 mL) was added to individual lipid film, and the final concentration of the cationic lipid was 1 mM. The mixtures were kept for hydration in deionized water at 4 °C for 12 h and then vortex-mixed for 5 min, followed by sonication of these suspensions for 15 min. The formulations were filtered through a 0.8-, 0.45- and 0.22-μm filter and stored at 4 °C under sterile conditions. 

### 3.4. Size Measurement and Zeta Potential Analysis of Liposome

The particle size distribution and zeta potential of the liposome were determined at room temperature by the dynamic light scanning method with the Delsa™ Nano C Particle Analyzer (Beckman Coulter). The cationic liposomes were prepared in deionized water as mentioned above. The concentration of cationic lipid in liposome was diluted to 0.33 μM by freshly deionized water. The particle size and the zeta potential were measured three times. Data were analyzed using a software package (ELS-Z software) supplied by the manufacturer (Beckman Coulter, Brea, CA, USA).

### 3.5. Gel Retardation Assay 

The gel retardation assay was performed for the sake of evaluating the binding interactions and optimizing the lipid-DNA (N/P) ratios between cationic lipid and DNA. Briefly, 0.8 μg of pEGFP-N1 was mixed with the liposome at various lipid/DNA charge ratios, including 1:2, 1:1, 2:1, 3:1, 4:1, 6:1. After 30 min of incubation, 20 μL of the DNA:lipid complex solution were mixed with 2 μL of 6× loading buffer and loaded onto a 1% agarose gel containing ethidium bromide. Electrophoresis was carried out for 45 min in 0.5× TBE running buffer solution at 100 V. The uncomplexed DNA moved out of the well, while the DNA/lipid complex still remained inside the well. The images were taken using a UV light illuminator.

### 3.6. Cell Culture

293T cells were cultured in Dulbecco’s Modified Eagle’s Medium (DMEM, Sigma) supplemented with 10% fetal bovine serum (FBS) in T25 culture flasks (Nunc, Denmark) and were incubated at 37 °C in a humidified atmosphere containing 5% CO_2_. Cells were passaged regularly by 0.1% trypsin (EDTA 0.02% and trypsin 0.1%) in PBS (pH 7.2).

### 3.7. In Vitro Transfection

#### 3.7.1. Fluorescence Microscopy

All transfection experiments were carried out in 293T cells in antibiotic-free media, unless otherwise specified. Before the transfection experiment, the cells were seeded into 24-well plates at a density of 1 × 10^5^ cells/well and incubated overnight before transfection. For transfection, the lipid formulation and DNA were serially diluted separately in Opti-MEM to reach a final volume of 50 μL. DNA was used at a concentration of 0.8 μg/well, unless otherwise specified. The lipid and DNA were complexed in a volume of 100 μL at room temperature for about 25 min. The lipid concentrations were varied to obtain the required lipid:DNA (N/P) charge ratios. Charge ratios here present the ratio of charge on the cationic lipid (mol) to nucleotide base molarity and were calculated by considering the average nucleotide mass of 330. After 25 min, 100 μL of media were added to the complexes (final DNA concentration, 12.12 μM). Then, the medium was discarded, and the cells were washed with DMEM. Then, the lipid-DNA complexes in 200 μL media were added to the cells. The plates were then incubated for 8 h at 37 °C in a humidified atmosphere containing 5% CO_2_. After the incubation, the lipid-DNA complexes were removed. Five hundred microliters of DMEM containing 10% FBS were added per well. Plates were further incubated for a period of 40 h before checking for the reporter gene expression. GFP expression was examined by fluorescence microscopy. Control groups were performed in each case by using commercially available transfection reagent, DC-Chol. All of the experiments were done in triplicate.

#### 3.7.2. Flow Cytometry

The reporter gene expression was quantified by flow cytometry at 48 h post-transfection. The percentage of transfected cells was obtained by determining the amount of the fluorescent-labeled cells, wherein non-transfected cells were used as the control. Approximately 1 × 10^5^ cells were analyzed to achieve the statistical data, which have been presented as the average. For flow cytometry analysis, 48 h post-transfection, the medium was removed from the wells. The cells were washed with PBS and trypsinized by adding 500 μL of 0.25% trypsin; then 500 μL PBS were added to each well, and the cells were collected. Triplicate cultures were analyzed by flow cytometry immediately using a Becton and Dickinson flow cytometer equipped with a fixed laser source at 488 nm.

### 3.8. Cytotoxicity

The toxicity of each cationic lipid formulation toward 293T cells in the absence of FBS was determined using the 3-(4,5-dimethylthiazole-2-yl)-2,5-diphenyl-tetrazolium bromide (MTT) reduction assay following literature procedures [37,38]. Nearly 1.5 × 10^4^ cells/well were plated in a 96-well plate. The concentration of liposome was set as 5–100 μmol. After 24 h, 20 μL of MTT solution were added, and the cells were incubated for another 3 h. The old media were removed, and 150 μL of DMSO were added per well. The reduced crystal violet was completely dissolved in DMSO, and then the absorbance of the solution was determined at 490 nm using a microtiter plate reader. Cell viability was calculated by the following equation:

[{A590treated cells − A590background}/{A590Untreated cells − A590 background}] × 100%

### 3.9. Statistical Analysis

Data are expressed as the means ± SD. Differences between experimental groups were evaluated by using the analysis of variance (ANOVA). The statistical significance of the difference between groups was evaluated by one-way ANOVA and the *t*-test. Values of *p* < 0.05 were accepted as significant.

## 4. Conclusions

In summary, two novel cationic lipids, **1a** and **1b**, were synthesized based on cholesterol and basic amino acids lysine and histidine. The effect of lysine or histidine residue head groups on the transfection efficiency was also investigated. The cationic lipids **1a** and **1b** exhibited quite different cytotoxicity and transfection efficiency when compared with commercially available DC-Chol, a liposome regent derived from the cholesterol backbone, which indicated that the biological properties of cationic lipids could possibly be controlled or optimized by choosing suitable head group and linker moieties. The newly synthesized cationic lipids, **1a** and **1b**, display sufficient transfection efficiency and less toxicity than DC-Chol. Among them, lipid **1a** displays more sufficient transfection efficiency than **1b** and DC-Chol, which make it a promising vector suitable for gene delivery.

## References

[1] Junquera E., Aicart E. (2014). Cationic lipids as transfecting agents of DNA in gene therapy. Curr. Top. Med. Chem..

[2] Jin L., Zeng X., Liu M., Deng Y., He N. (2014). Current progress in gene delivery technology based on chemical methods and nano-carriers. Theranostics.

[3] Anderson W.F. (1998). Human gene therapy. Nature.

[4] Islam M.A., Firdous J., Choi Y.J., Yun C.H., Cho C.S. (2014). Regulation of endocytosis by non-viral vectors for efficient gene activity. J. Biomed. Nanotechnol..

[5] Ronsin G., Perrin C., Guedat P., Kremer A., Camilleri P., Kirby A.J. (2001). Novel spermine-based cationic gemini surfactants for gene delivery. Chem. Commun..

[6] Raper S.E., Yudkoff M., Chirmule N., Gao G.P., Nunes F., Haskal Z.J., Furth E.E., Propert K.J., Robinson M.B., Magosin S. (2002). A pilot study of *in vivo* liver-directed gene transfer with an adenoviral vector in partial ornithine transcarbamylase deficiency. Hum. Gene. Ther..

[7] Wiethoff C.M., Koe J.G., Koe G.S., Middaugh C.R. (2004). Compositional effects of cationic lipid/DNA delivery systems on transgene expression in cell culture. J. Pharm. Sci..

[8] Marshall E. (2002). Gene therapy. What to do when clear success comes with an unclear risk?. Science.

[9] Mintzer M.A., Simanek E.E. (2009). Nonviral vectors for gene delivery. Chem. Rev..

[10] Sen J., Chaudhuri A. (2005). Design, syntheses, and transfection biology of novel non-cholesterol-based guanidinylated cationic lipids. J. Med. Chem..

[11] Suh M.S., Shim G., Lee H.Y., Han S.E., Yu Y.H., Choi Y., Kim K., Kwon I.C., Weon K.Y., Kim Y.B., Oh Y.K. (2009). Anionic amino acid-derived cationic lipid for siRNA delivery. J. Control. Release.

[12] Li L., Song H., Luo K., He B., Nie Y., Yang Y., Wu Y., Gu Z. (2011). Gene transfer efficacies of serum-resistant amino acids-based cationic lipids: Dependence on headgroup, lipoplex stability and cellular uptake. Int. J. Pharm..

[13] Sheng R., Luo T., Li H., Sun J., Wang Z., Cao A. (2013). “Click” synthesized sterol-based cationic lipids as gene carriers, and the effect of skeletons and headgroups on gene delivery. Bioorg. Med. Chem..

[14] Bajaj A., Kondiah P., Bhattacharya S. (2007). Design, synthesis, and *in vitro* gene delivery efficacies of novel cholesterol-based gemini cationic lipids and their serum compatibility: A structure-activity investigation. J. Med. Chem..

[15] Chien P.Y., Wang J., Carbonaro D., Lei S., Miller B., Sheikh S., Ali S.M., Ahmad M.U., Ahmad I. (2005). Novel cationic cardiolipin analogue-based liposome for efficient DNA and small interfering RNA delivery *in vitro* and *in vivo*. Cancer Gene Ther..

[16] Lv H., Zhang S., Wang B., Cui S., Yan J. (2006). Toxicity of cationic lipids and cationic polymers in gene delivery. J. Control. Release.

[17] Pungente M.D., Jubeli E., Opstad C.L., Al-Kawaz M., Barakat N., Ibrahim T., Abdul K.N., Raju L., Jones R., Leopold P.L. (2012). Synthesis and preliminary investigations of the siRNA delivery potential of novel, single-chain rigid cationic carotenoid lipids. Molecules.

[18] Egilmez N.K., Iwanuma Y., Bankert R.B. (1996). Evaluation and optimization of different cationic liposome formulations for *in vivo* gene transfer. Biochem. Biophys. Res. Commun..

[19] Farhood H., Gao X., Barsoum J., Huang L. (1995). Codelivery to mammalian cells of a transcriptional factor with cis-acting element using cationic liposomes. Anal. Biochem..

[20] Gao X., Huang L. (1991). A novel cationic liposome reagent for efficient transfection of mammalian cells. Biochem. Biophys. Res. Commun..

[21] Kim B.K., Seu Y.B., Bae Y.U., Kwak T.W., Kang H., Moon I.J., Hwang G.B., Park S.Y., Doh K.O. (2014). Efficient delivery of plasmid DNA using cholesterol-based cationic lipids containing polyamines and ether linkages. Int. J. Mol. Sci..

[22] Kim B.K., Doh K.O., Nam J.H., Kang H., Park J.G., Moon I.J., Seu Y.B. (2009). Synthesis of novel cholesterol-based cationic lipids for gene delivery. Bioorg. Med. Chem. Lett..

[23] Okuda T., Sugiyama A., Niidome T., Aoyagi H. (2004). Characters of dendritic poly(l-lysine) analogues with the terminal lysines replaced with arginines and histidines as gene carriers *in vitro*. Biomaterials.

[24] Karmali P.P., Kumar V.V., Chaudhuri A. (2004). Design, syntheses and in vitro gene delivery efficacies of novel mono-, di- and trilysinated cationic lipids: A structure-activity investigation. J. Med. Chem..

[25] Karmali P.P., Majeti B.K., Sreedhar B., Chaudhuri A. (2006). *In vitro* gene transfer efficacies and serum compatibility profiles of novel mono-, di-, and tri-histidinylated cationic transfection lipids: A structure-activity investigation. Bioconjug. Chem..

[26] Shigeta K., Kawakami S., Higuchi Y., Okuda T., Yagi H., Yamashita F., Hashida M. (2007). Novel histidine-conjugated galactosylated cationic liposomes for efficient hepatocyte-selective gene transfer in human hepatoma HepG2 cells. J. Control. Release.

[27] Verma S.K., Mani P., Sharma N.R., Krishnan A., Kumar V.V., Reddy B.S., Chaudhuri A., Roy R.P., Sarkar D.P. (2005). Histidylated lipid-modified Sendai viral envelopes mediate enhanced membrane fusion and potentiate targeted gene delivery. J. Biol. Chem..

[28] Sheng R., Luo T., Li H., Sun J., Wang Z., Cao A. (2014). Cholesterol-based cationic lipids for gene delivery: contribution of molecular structure factors to physico-chemical and biological properties. Colloids Surf. B Biointerfaces.

[29] Ghosh Y.K., Visweswariah S.S., Bhattacharya S. (2002). Advantage of the ether linkage between the positive charge and the cholesteryl skeleton in cholesterol-based amphiphiles as vectors for gene delivery. Bioconjug. Chem..

[30] Ghosh Y.K., Visweswariah S.S., Bhattacharya S. (2000). Nature of linkage between the cationic headgroup and cholesteryl skeleton controls gene transfection efficiency. Febs. Lett..

[31] Angelini G., Pisani M., Mobbili G., Marini M., Gasbarri C. (2013). Neutral liposomes containing crown ether-lipids as potential DNA vectors. Biochim. Biophys. Acta.

[32] Choi J.S., Lee E.J., Jang H.S., Park J.S. (2001). New cationic liposomes for gene transfer into mammalian cells with high efficiency and low toxicity. Bioconjug. Chem..

[33] Liu D., Hu J., Qiao W., Li Z., Zhan S., Cheng L. (2005). Synthesis and characterization of a series of carbamate-linked cationic lipids for gene delivery. Lipids.

[34] Felgner J.H., Kumar R., Sridhar C.N., Wheeler C.J., Tsai Y.J., Border R., Ramsey P., Martin M., Felgner P.L. (1994). Enhanced gene delivery and mechanism studies with a novel series of cationic lipid formulations. J. Biol. Chem..

[35] Zhang Y., Li H., Sun J., Gao J., Liu W., Li B., Guo Y., Chen J. (2010). DC-Chol/DOPE cationic liposomes: A comparative study of the influence factors on plasmid pDNA and siRNA gene delivery. Int. J. Pharm..

[36] Zimmer A., Aziz S.A., Gilbert M., Werner D., Noe C.R. (1999). Synthesis of cholesterol modified cationic lipids for liposomal drug delivery of antisense oligonucleotides. Eur. J. Pharm. Biopharm..

[37] Mosmann T. (1983). Rapid colorimetric assay for cellular growth and survival: application to proliferation and cytotoxicity assays. J. Immunol. Methods.

[38] Hansen M.B., Nielsen S.E., Berg K. (1989). Re-examination and further development of a precise and rapid dye method for measuring cell growth/cell kill. J. Immunol. Methods.

